# Tumor Treating Fields (TTFields) combined with the drug repurposing approach CUSP9v3 induce metabolic reprogramming and synergistic anti-glioblastoma activity in vitro

**DOI:** 10.1038/s41416-024-02608-8

**Published:** 2024-02-23

**Authors:** Qiyu Cao, Annika Hajosch, Richard Eric Kast, Christopher Loehmann, Michal Hlavac, Pamela Fischer-Posovszky, Hannah Strobel, Mike-Andrew Westhoff, Markus D. Siegelin, Christian Rainer Wirtz, Marc-Eric Halatsch, Georg Karpel-Massler

**Affiliations:** 1 Department of Neurosurgery, Ulm University Medical Center, Ulm, Germany; 2 IIAIGC Study Center, Burlington, VT USA; 3 Department of Pediatrics and Adolescent Medicine, Ulm University Medical Center, Ulm, Germany; 4 Department of Pathology, Columbia University Irving Medical Center, New York, NY USA; 5 Department of Neurosurgery, Cantonal Hospital of Winterthur, Winterthur, Switzerland

**Keywords:** Cancer metabolism, CNS cancer

## Abstract

**Background:**

Glioblastoma represents a brain tumor with a notoriously poor prognosis. First-line therapy may include adjunctive Tumor Treating Fields (TTFields) which are electric fields that are continuously delivered to the brain through non-invasive arrays. On a different note, CUSP9v3 represents a drug repurposing strategy that includes 9 repurposed drugs plus metronomic temozolomide. Here, we examined whether TTFields enhance the antineoplastic activity of CUSP9v3 against this disease.

**Methods:**

We performed preclinical testing of a multimodal approach of TTFields and CUSP9v3 in different glioblastoma models.

**Results:**

TTFields had predominantly synergistic inhibitory effects on the cell viability of glioblastoma cells and non-directed movement was significantly impaired when combined with CUSP9v3. TTFields plus CUSP9v3 significantly enhanced apoptosis, which was associated with a decreased mitochondrial outer membrane potential (MOMP), enhanced cleavage of effector caspase 3 and reduced expression of Bcl-2 and Mcl-1. Moreover, oxidative phosphorylation and expression of respiratory chain complexes I, III and IV was markedly reduced.

**Conclusion:**

TTFields strongly enhance the CUSP9v3-mediated anti-glioblastoma activity. TTFields are currently widely used for the treatment of glioblastoma patients and CUSP9v3 was shown to have a favorable safety profile in a phase Ib/IIa trial (NCT02770378) which facilitates transition of this multimodal approach to the clinical setting.

## Introduction

Glioblastoma represents a malignant brain tumor that develops from the intrinsic components of the brain [1]. Despite aggressive first-line therapy that includes maximal safe resection and radio-/chemotherapy with temozolomide, patients with this disease face a dismal prognosis due to therapeutic resistance and recurrence of the tumor [2]. More recently, in a phase III randomized, controlled clinical trial (NCT00916409, EF-14), patients with newly-diagnosed glioblastoma were stratified to either receive radio-/chemotherapy according to the EORTC protocol alone or concomitant with TTFields. The addition of TTFields to maintenance temozolomide was shown to extend the median progression-free survival from 4 to 6.7 months and the median overall survival from 16 to 20.9 months [3]. As a consequence, TTFields joined forces for the first-line therapy of patients with newly-diagnosed glioblastoma.

TTFields involve the transcutaneous delivery of continuous low-intensity (1–3 V/cm), intermediate-frequency (100–500 kHz) alternating electric fields that exert biophysical forces on charged and polarizable molecules [4]. In multiple clinical studies, TTFields were shown to have a favorable safety profile and low side effect burden [5]. With respect to the mechanism of action, the anti-mitotic effects of TTFields are most widely recognized [6–8]. More recently, interference with DNA repair [9, 10] and pro-immunogenic effects [11–13] were observed. Moreover, TTFields were reported to increase the cell permeability of cancer cells [14, 15] and to promote the penetration of small molecules across the blood brain barrier [16].

Intratumoral heterogeneity is regarded as a core feature of glioblastoma and is in great part responsible for the incalcitrant nature of this disease [17]. As a consequence, non-predictable clonal diversion and plasticity of the intracellular signaling pathways in response to changes in the microenvironment and therapeutic pressure render a single-drug/single-target approach futile. To address this, the CUSP9 strategy was developed as a fusion of multitargeting and drug repurposing strategies [18, 19]. A drug repurposing approach was chosen to accelerate clinical transition, minimizing financial costs and safety risks [20]. CUSP9v3 includes nine drugs that are already approved for other indications but also have anti-glioblastoma activity and which are combined with metronomic temozolomide. In vitro, CUSP9v3 was shown to have a strong antineoplastic activity in the setting of glioblastoma [21]. Moreover, in a phase Ib/IIa clinical trial (NCT02770378), a favorable safety profile and good tolerability was found [22].

In this study, we performed preclinical testing of a novel therapeutic strategy combining TTFields and the drug repurposing strategy CUSP9v3 in multiple in vitro models of glioblastoma. Our data show for the first time that this combination treatment leads to predominantly synergistic antineoplastic activity in this setting. TTFields concomitant with CUSP9v3 induce an enhanced pro-apoptotic response which goes along with suppression of oxidative phosphorylation and downregulation of several complexes of the respiratory chain. Both treatment modalities on their own have been safely used in the clinical setting thus facilitating a rapid clinical translation of this combined approach.

## Materials and methods

### Reagents

Aprepitant (Selleckchem, Houston, TX, USA; stock: 100 mg/mL), auranofin (Sigma Aldrich, St. Louis, MO, USA; stock: 5 mg/mL), celecoxib (Sigma Aldrich; stock: 20 mg/mL), disulfiram (Sigma Aldrich; stock: 5 mg/mL), itraconazole (Abcam, Cambridge, UK; stock: 20 mg/mL) and ritonavir (Sigma Aldrich; stock: 10 mg/mL) were dissolved in dimethyl sulfoxide (DMSO). Captopril (Sigma Aldrich; stock: 50 mg/mL), minocycline (Sigma Aldrich; stock: 25 mg/mL) and sertraline (Sigma Aldrich; stock: 3.8 mg/mL) were dissolved in H_2_O. All stock solutions were stored at −20 °C. For all experiments, final concentrations of DMSO were below 0.1% (*v*/*v*). Table 1 summarizes the concentrations for each component at the respective dilution used.

**Table 1:** Concentration of the CUSP9v3 compounds in different preparations.

Compound	Stock (mg/ml)	Solvent	1/2.5 (µg/ml)	1/5 (µg/ml)	1/7.5 (µg/ml)	1/10 (µg/ml)
Aprepitant	100	DMSO	0.4	0.2	0.1333	0.1
Auranofin	5	DMSO	0.36	0.18	0.12	0.09
Captopril	50	H_2_O	0.52	0.26	0.1733	0.13
Celecoxib	20	DMSO	0.72	0.36	0.24	0.18
Disulfiram	5	DMSO	0.02	0.01	0.0067	0.005
Itraconazole	20	DMSO	0.1968	0.0984	0.0656	0.0492
Minocycline	25	H_2_O	1.0	0.5	0.3333	0.25
Ritonavir	10	DMSO	4.924	2.462	1.6413	1.231
Sertraline	3.8	H_2_O	0.012	0.006	0.004	0.003

### In vitro application of TTFields

TTFields were applied using the Inovitro^TM^ Lab Bench System (Novocure^TM^, Haifa, Israel) as described before [23]. For all experiments, the cells were treated with TTFields at an intensity of 1.46 V/cm (RMS) and a frequency of 200 kHz.

### Cell cultures and growth conditions

U251 human glioblastoma cells were purchased from Sigma Aldrich in January 2017. The identity of these cells was confirmed by Sigma Aldrich. The initial stocks were expanded, frozen and stored in liquid nitrogen. Fresh aliquots were thawed every 6 weeks. Primary cultured human glioblastoma cells PC38, PC40 and PC128 as well as SC38, SC40 and SC128 stem‐like glioblastoma cells were obtained from tumor resections at our institution [24, 25]. The cells were cultured as previously described [26, 27]. The procedures were approved by the institutional review board of the University of Ulm (No.162/10). Consent was granted by the patients or next of kin.

### MTT assay

In order to examine effects on cell viability, 3-(4, 5-dimethylthiazol-2-yl)-2, 5-diphenyltetrazolium bromide (MTT) assays were performed. 5 × 10^4^ cells/well were seeded in 6-well flat-bottomed plates which had a glass coverslip put inside. The cells were left overnight at 37 °C to attach to the coverslips prior to changing the medium to DMEM supplemented with 1.5% FBS and the respective treatments. After 72 h, the medium was aspirated and 800 µL MTT solution were added to the wells followed by incubation at 37 °C for 3 h. The reaction was stopped by adding 800 µL of 100% isopropanol (Sigma Aldrich) and optical densities were measured at 570 nm using an automated microplate reader.

### Spheroid assay

Spheroids were generated to assess effects of the combination treatment in 3 dimensions as described before [26]. Briefly, 3.5 × 10^4^ U251 or 0.75 × 10^4^ PC40 and PC128 cells were resuspended in 20 µL of a mixture of 80% Matrigel/20% DMEM prior to incubation for 1 h at 37 °C. Then, the cell/Matrigel matrix was gently transferred to 12-well plates containing DMEM supplemented with 10% FBS (20% FBS for PC128 cells) and the spheroids were allowed to form over a period of 5 d. Afterwards, they were transferred again to 12-well plates containing DMEM supplemented with 1.5% FBS and the respective treatments or solvent. Treatments were repeated on day 7 and day 9. On day 12, microphotographs were taken at 4 × magnification and maximal sphere expansion (A) was calculated using the formula $A=\pi {r}^{2}$. In addition, CellTiter-Glo^®^ (Roche Diagnostics, Indianapolis, IN, USA) assays were used to assess antiproliferative effects on day 12. To this purpose, spheroids suspended in 100 µL of medium were transferred to opaque-walled 96-well plates prior to adding 100 µL of the CellTiter-Glo^®^ solution and incubation for 10 min at RT. Afterwards, luminescence was measured.

### Measurement of apoptosis

Apoptosis was detected using annexin V/propidium iodide (PI) or PI staining followed by flow cytometry as described before [26, 28].

For PI staining, 5 × 10^4^ cells were seeded in 6-well plates and allowed to attach overnight. After the respective treatments, the supernatants and enzymatically detached cells (Trypsin/EDTA, Biochrom AG, Berlin, Germany) were centrifuged for 5 min at 1800 rpm prior to washing twice with PBS. Then, 200 μl PI staining solution containing 0.05% trisodiumcitrate-dihydrate (Carl Roth, Karlsruhe, Germany), 0.05% triton-X100 and 0.05 mg/ml PI (Sigma Aldrich) was added and cells were incubated for 30 min at 4 °C prior to flow cytometric analysis.

For Annexin V/PI staining, 5 × 10^4^ cells were seeded in 6-well plates and allowed to attach overnight. After the respective treatments, the supernatants and enzymatically detached cells (Trypsin/EDTA) were collected. Next, the cells were centrifuged and washed twice with ice-cold annexin V binding buffer containing 140 mM NaCl, 2.5 mM Ca^2+^ and 1 M 4-(2-hydroxyethyl) piperazine-1- ethane sulfonic acid (pH 7.4) followed by resuspension in 100 μl binding buffer and incubation with 2.5 μl Annexin-V-FLUOS (Roche Diagnostics) for 15 min at RT. Then, the cells were washed once with ice-cold annexin V binding buffer prior to resuspension in 300 μl buffer, after which PI was added to a final concentration of 2.5 μM right before performing each single measurement. For each flow cytometric analysis, 10,000 events were recorded using a FACS Canto™ II flow cytometer (BD Biosciences, NJ, USA). FlowJo software version 10.5.3 (Tree Star, Ashland, OR, USA) was used for further quantitative analyses.

To detect intrinsic apoptosis, staining with MitoTracker^TM^ (Roche Diagnostics) was performed. To this purpose, 5 × 10^4^ cells were seeded in 6-well plates and allowed to attach overnight. Following the defined treatments and treatment periods, cell culture plates were centrifuged at 1300 rpm for 5 min prior to aspirating the supernatant and adding 200 nM MitoTracker^TM^ staining solution. The cells were incubated for 20 min at 37 °C. Next, the cells were enzymatically detached and centrifuged for 5 min at 1800 rpm. The supernatants were discarded and the cells were resuspended in 300 μl PBS followed by flow cytometric analysis. 10,000 events per condition were recorded by a FACS Canto™ II flow cytometer (BD Biosciences) and the data analysis was performed with FlowJo 10.5.3 (Tree Star).

### Western blot analysis

Protein expression was determined by Western blot analysis as previously reported [28], using the following primary antibodies: rabbit anti-Mcl-1 (1:1000; #5453 S, clone: D35A5, CST Cell Signaling Technology, Danvers, MA, U.S.A.), rabbit anti-Bcl-2 (1:1000; #2872, CST), rabbit anti-Bcl-xL (1:500; #2764, clone 54H6, CST), rabbit anti-human caspase-3 (1:1000; #9662, CST), total OXPHOS human WB antibody cocktail (1:1000; #ab110411, Abcam, Cambridge, U.K.) and mouse anti-human β-actin (1:2000; clone: AC15; Sigma Aldrich). Secondary HRP-linked antibodies were purchased from CST (#7076 S, #7074 S).

### cDNA synthesis and real-time quantitative PCR

Real time PCR experiments were performed as previously described [27] using the primers as outlined in Table 2.

**Table 2:** Primer sequences.

Gene	Forward sequence	Reverse sequence
Bcl-2	ATC GCC CTG TGG ATG ACT GAG T	GCC AGG AGA AAT CAA ACA GAG GC
GAPDH	GTC TCC TCT GAC TTC AAC AGC G	ACC ACC CTG TTG CTG TAG CCA A

### Cell migration assays

Effects on cell migration were analyzed by scratch assays and time-lapse live cell microscopy imaging.

For scratch assays, 5 × 10^4^ cells/well (PC38 and PC40) or 9 × 10^4^ cells/well (U251) were seeded on 6-well plates containing glass cover slip inserts. After 24 h, the coverslips were transferred to TTFields ceramic dishes followed by 24 h of the respective treatments. Then, the coverslips were transferred to 12-well plates and a 200 μl pipet tip was used to induce straight scratches across subconfluent cell layers. Sequential microscopic images were taken at defined time points at 10 × magnification (Carl Zeiss, Primovert/AxioCam ICc1, Oberkochen, Germany). The area of the scratch was analyzed using the NIH ImageJ software (http://imagej.nih.gov/ij).

For time-lapse live cell microscopy imaging, 5 × 10^4^ (PC128 and PC40) or 9 × 10^4^ (U251) cells were seeded on glass cover inserts placed in 6-well plates and allowed to attach overnight. Then, the cover slips were transferred to TTFields ceramic dishes and treated for 24 h. Afterwards, the cells were enzymatically detached and 3 × 10^3^ cells/well were seeded on 96-well plates, and microscopic images were taken with an Incucyte® S3 live-cell analysis system (Sartorius/Essen BioScience) every 30 min for a total observation time of 24 h. During this period, cells were kept at standard culture conditions (37 °C, 5% CO_2_, water-saturated atmosphere). Single-cell tracking was perfomed with the MtrackJ plugin (www.imagescience.org/meijering/software/mtrackj/) for the NIH ImageJ software. Normalized “wind-rose” plots were generated with the chemotaxis and migration tool from Integrated BioDiagnostics (Martinsried, Germany, www.ibidi.com).

### Extracellular flux analysis

5 × 10^4^ PC128 or 9 × 10^4^ U251 cells were seeded on glass cover inserts placed in 6-well plates and allowed to attach overnight. Then, the cover slips were transferred to TTFields ceramic dishes and treated for 24 h. Afterwards, the cells were enzymatically detached and 1 × 10^4^ cells/well were seeded on XF96 V3 PS cell culture microplates (Agilent Technologies Inc., Wilmington, DE, USA) and allowed to attach for 24 h followed by one wash with XF assay medium containing 5 mM glucose (pH adjusted to 7.5). Afterwards, the mito stress test kit (Agilent Technologies Inc.) was used as described by the manufacturer applying serial injections of oligomycin at a final concentration of 2 µM, FCCP at a final concentration of 2 µM and rotenone/antimycin A at a final concentration of 0.5 µM. All analyses were performed on an Agilent Seahorse XFe96 analyzer.

### Statistical analysis

Statistical significance was determined by one-way ANOVA followed by Newman–Keuls post hoc analysis using PRISM version 5.04 (GraphPad, La Jolla, CA, U.S.A.). Statistical significance was assumed for *p* < 0.05. BLISS analysis was performed to characterize the interaction between the different treatment modalities [29, 30]. The total response was calculated as a fractional response to treatment modality A (F_a_) + fractional response to treatment modality B (F_b_) - F_a_ × F_b_. A ratio of the actual total response and the expected total response of 0.9 to 1.1 was considered as additive, a ratio <0.9 as antagonistic and a ratio >1.1 as synergistic.

## Results

### TTFields concomitant with CUSP9v3 reduce the viability of glioblastoma cells in a predominantly synergistic manner

**Fig. 1:**
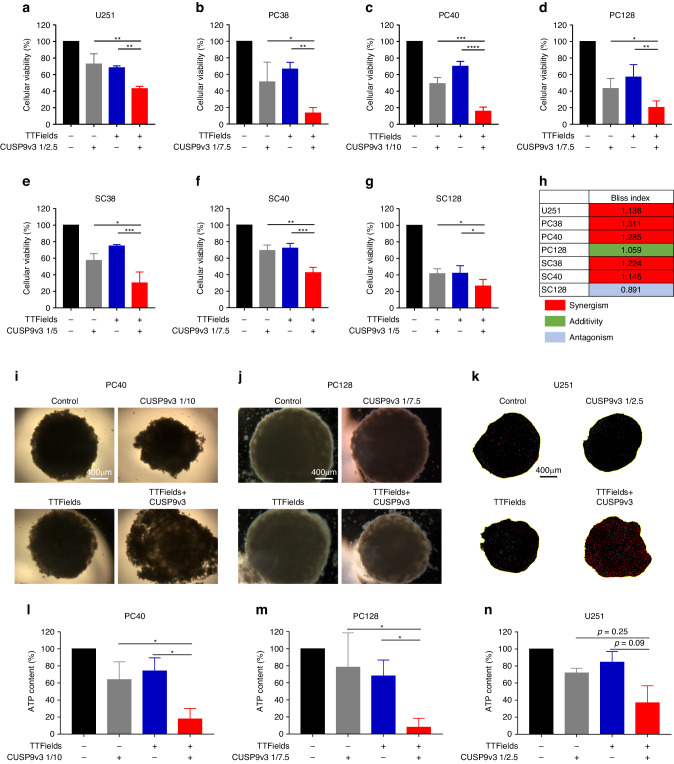
TTFields combined with CUSP9v3 have a synergistic inhibitory effect on the cell viability of glioblastoma cells. Established (**a**), primary cultured (**b**–**d**) and stem‐like (**e**–**g**) glioblastoma cells were treated for 72 h as indicated. Cell viability was determined by MTT assays. Data are presented as mean and SD from at least three independent experiments. *p < 0.05, **p < 0.01, ***p < 0.005. **h** BLISS analysis was performed for cells treated as described for (**a**–**g**). A synergistic interaction is highlighted in red; an additive interaction is highlighted in green and an antagonistic interaction is highlighted in blue. Data are representative for three independent experiments. **i**, **j** Microphotographs of PC40 and PC128 spheroids that were grown for 12 d. Treatment as indicated was performed on d5, d7 and d9. Magnification: × 4. **k** Representative microphotographs of U251 spheroids treated as described for (**i**, **j**) and stained with propidium iodide prior to fluorescent imaging (Cytation5 cell imaging multimode reader and Gen5 software, Agilent BioTek, Waldbronn, Germany). Magnification: × 4. **l**–**n** PC40, PC128 and U251 spheroids were treated as described for (**i**, **j**). CellTiter-Glo® assays were performed to determine the ATP content. Columns: mean. Error bars: SD. *N* = 3. *p < 0.05, **p < 0.01.

Both TTFields and CUSP9v3 have been previously shown to affect the viability of glioblastoma cells when applied alone. We hypothesized that an application of both treatment modalities results in a synergistic inhibitory effect on the viability of glioblastoma cells. In order to test this hypothesis, U251 cells (Fig. 1a), PC38, PC40 and PC128 glioblastoma primary cultures (Fig. 1b–d) as well as SC38, SC40 and SC128 stem-like glioblastoma cells (Fig. 1e–g) were treated with solvents, TTFields, CUSP9v3 or both modalities. After 72 h of treatment MTT assays were performed. In all glioblastoma models tested, treatment with either TTFields or CUSP9v3 alone led to reduced cell viability (Fig. 1a–g). However, when applied together, the inhibitory effect on cell viability was significantly enhanced across all cells tested including those with stem-like features (Fig. 1a–g). To further characterize the nature of the interaction of the two treatment modalities, Bliss analysis was performed. As shown in Fig. 1h, for the majority of the cells, a synergistic interaction between TTFields and CUSP9v3 was found. In PC128 cells, an additive interaction was found and only in SC128 cells, an antagonistic effect of the combination was observed (Fig. 1h).

### TTFields concomitant with CUSP9v3 inhibit the growth of spheroids

Considering that patients’ tumors grow spatially in 3 dimensions, we sought to determine whether TTFields and CUSP9v3 would be able to impair the proliferation of tumor cells in a more representative setting by using 3-dimensional spheroid assays. PC40, PC128 and U251 spheroids were allowed to form over a period of 5 d prior to starting with the treatments for another 7 d. Both TTFields and CUSP9v3 alone caused a slight inhibition of spheroid growth (Fig. 1i, j, l–n and Supplementary Fig. 1). In spheroids that were derived from U251 and PC128, this inhibitory effect was more pronounced when TTFields and CUSP9v3 were used together as shown in Fig. 1j and Supplementary Fig. 1. While the combination treatment did not lead to a reduction in size of spheroids derived from PC40 cells (Supplementary Fig. 1), we did observe a reduced cellular density and also a decreased cohesion of the cells within the spheroids (Fig. 1i). These observations were confirmed by quantitative analysis showing a significantly reduced ATP content when compared to treatment with either TTFields or CUSP9v3 alone in PC40 and PC128 cells (Fig. 1l, m). In U251 cells, a marked decrease of the ATP content following the dual treatment was also noticed (Fig. 1n). However, statistical significance was not reached.

### TTFields together with CUSP9v3 lead to enhanced apoptosis

**Fig. 2:**
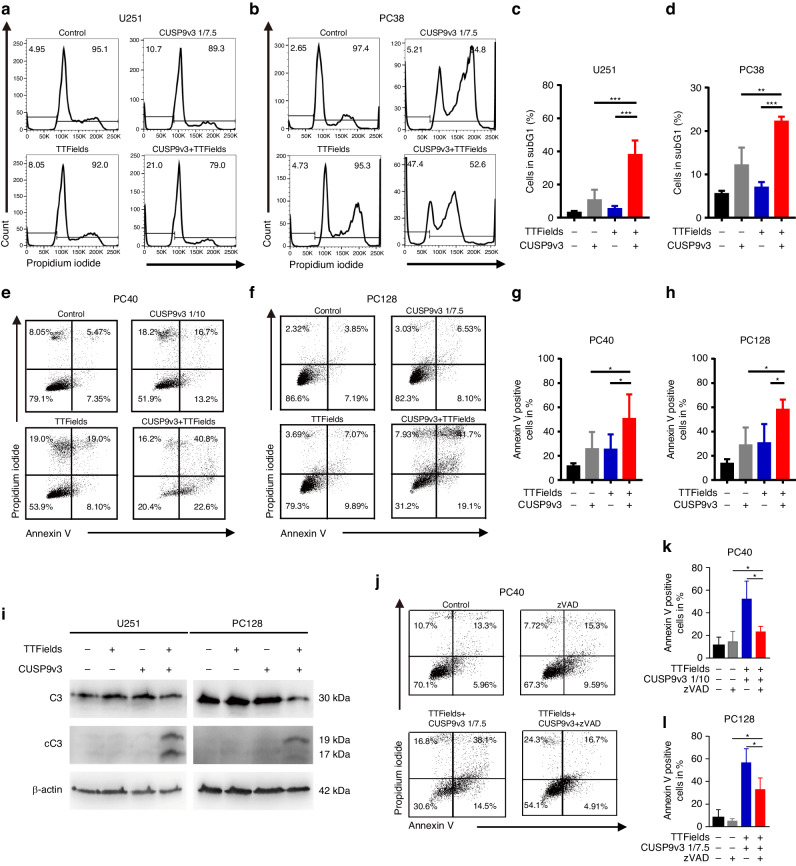
Combined treatment with TTFields and CUSP9v3 induces caspase-dependent apoptosis. **a**, **b** Representative flow plots of U251 and PC38 cells treated as indicated for 48 h under serum starvation (1.5% FBS). Staining with Propidium iodide and flow cytometry was performed. **c**, **d** Quantitative representation of U251 and PC38 cells subjected to the same treatment as outlined in (**a**, **b**). Columns: mean. Error bars: SD. *N* = 3. **p < 0.01, ***p 0.005. **e**, **f** Representative flow plots of PC40 and PC128 cells subjected to indicated treatments for 48 h. Staining with Annexin V and Propidium iodide was done prior to flow cytometry. **g**, **h** Quantitative representation of PC40 and PC128 cells treated as described for (**e**, **f**). Columns: mean. Error bars: SD. *N* = 3. *p < 0.05. **i** U251 and PC128 cells were treated as indicated for 24 h. Whole cell extracts were collected. Western blot analysis was performed for caspase 3 (C3) and cleaved caspase 3 (cC3) expression. Expression of β-actin was used to control for loading. **j** Representative flow plots of PC40 cells treated for 24 h with CUSP9v3 plus TTFields in the presence or absence of 30 µM zVAD.fmk. Staining with Annexin V/Propidium iodide followed by flow cytometry was performed. **k**, **l** Quantitative representation of PC40 and PC128 cells treated as described for (**j**). Columns: mean. Error bars: SD. *N* = 3. *p < 0.05.

To address the question of whether the inhibitory effect of the combination treatment on cell viability and the growth of spheroids is due to induction of cell death, we stained U251 spheroids with PI (Fig. 1k). Spheroids that were subjected to treatment with TTFields and CUSP9v3 showed a strong PI staining which was markedly less pronounced in those spheroids that were treated with either TTFields or CUSP9v3 alone. In line with this finding, the subG1 fraction of U251 and PC38 cells that were treated with PI and analyzed by flow cytometry was significantly increased following dual treatment with TTFields and CUSP9v3 (Fig. 2a–d). In order to further dissect whether this observation can be interpreted as a result of apoptosis, PC40 and PC128 cells were stained with Annexin V and PI followed by flow cytometric analysis. As shown in Fig. 2e–h, glioblastoma cells subjected to treatment with the combination of CUSP9v3 and TTFields showed a significant increase of the fraction of Annexin V‐positive (apoptotic) cells when compared to either treatment alone.

### The pro-apoptotic effect of dual treatment with TTFields and CUSP9v3 is caspase-dependent

**Fig. 3:**
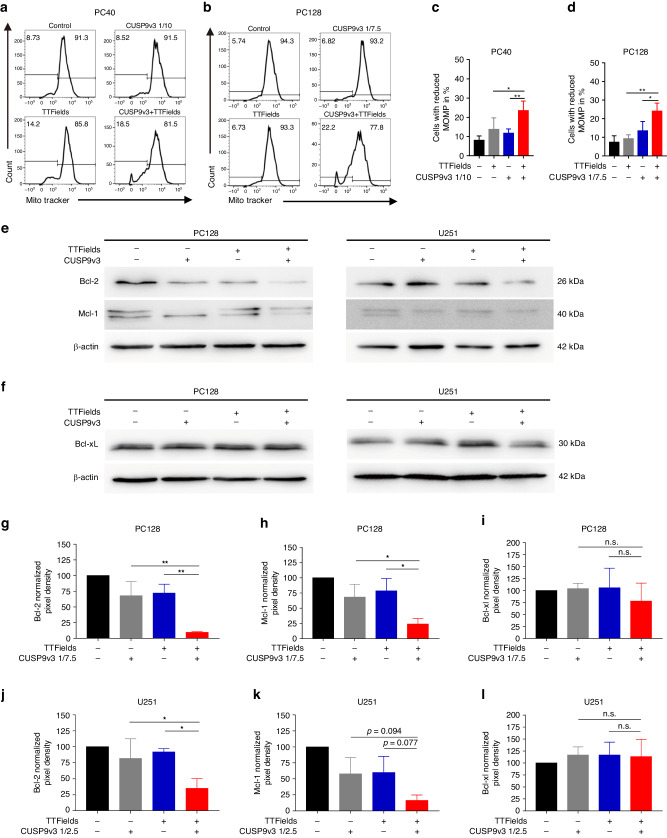
The simultaneous treatment with TTFields and CUSP9v3 suppresses expression of Bcl-2 and Mcl-1. **a**, **b** Representative histograms of PC40 and PC128 cells that were treated as indicated for 48 h prior to staining with MitoTracker^TM^ and flow cytometric analysis. **c**, **d** Quantitative representation of the fraction of cells treated as described for (**a**, **b**) showing reduced mitochondrial outer membrane potential (MOMP). Columns: mean. Error bars: SD. *N* = 3. *p < 0.05, **p < 0.01. **e** PC128 and U251 cells were treated for 24 h with TTFields and CUSP9v3 (1/7.5 for PC128 and 1/2.5 for U251) or solvent. Whole cell extracts were collected and the expression of Bcl-2 and Mcl-1 was determined by Western blot analysis. β-actin served as loading control. **f** PC128 and U251 cells were treated as described for (**e**). Whole cell extracts were collected and the expression of Bcl-xL was determined by Western blot analysis. β-actin served as loading control. **g**–**l** Quantitative representation of PC128 and U251 cells treated as described for (**e**, **f**). Densitometric analysis was performed using the Bio-1D software (Vilber Lourmat, Eberhardzell, Germany). Data were normalized to the corresponding β-actin signal and control. Columns: mean. Error bars: SD. *N* = 3. *p < 0.05, **p < 0.01. n.s.: non-significant.

Since we observed a TTFields/CUSP9v3-induced pro-apoptotic response as outlined by an increased fraction of Annexin V-positive cells (Fig. 2e, f), we decided next to examine whether this effect is caspase-dependent. While treatment with TTFields or CUSP9v3 alone did not induce cleavage of caspase-3 beyond baseline levels, the dual treatment led to a markedly enhanced cleavage of effector caspase-3 (Fig. 2i). Along this line, the pro-apoptotic response of the combination was significantly reduced when PC40 and PC128 cells were treated with TTFields/CUSP9v3 in the presence of the pan-caspase inhibitor zVAD (Fig. 2j–l). Next, we investigated whether the pro-apoptotic effect is in part mediated by the intrinsic pathway. To this end, PC40 and PC128 glioblastoma cells were treated with CUSP9v3, TTFields or both. Staining with MitoTracker^TM^ was performed to assess effects on the mitochondrial membrane potential (Fig. 3a–d). In both primary cultures tested, the dual treatment led to a significant increase in the fraction of cells with a reduced mitochondrial outer membrane potential.

### Concomitant treatment with TTFields and CUSP9v3 suppresses the expression of Bcl-2 and Mcl-1

Given that the dual treatment reduces the mitochondrial outer membrane potential, we sought to examine the effect on the expression of anti-apoptotic Bcl-2 family proteins which are important regulators of the intrinsic pathway of apoptosis. While in PC128 cells treatment with either TTFields or CUSP9v3 alone led to decreased expression of Bcl-2, only a slight decrease in Bcl-2 levels was observed in U251 cells (Fig. 3e, g, j). However, in both PC128 and U251 cells treatment with either TTFields or CUSP9v3 alone led to downregulation of Mcl-1 (Fig. 3e, h, k). Notably, treatment with TTFields and CUSP9v3 together markedly reduced the expression of Bcl-2 and Mcl-1 when compared to treatment with either modality alone (Fig. 3e, g, h, j, k). The anti-apoptotic Bcl-2 family protein Bcl-xL was not affected by TTFields or CUSP9v3 alone or when applied together (Fig. 3f, i, l).

### Concomitant treatment with TTFields and CUSP9v3 enhances the turnover of Mcl-1 protein and downregulates Bcl-2 mRNA transcripts

**Fig. 4:**
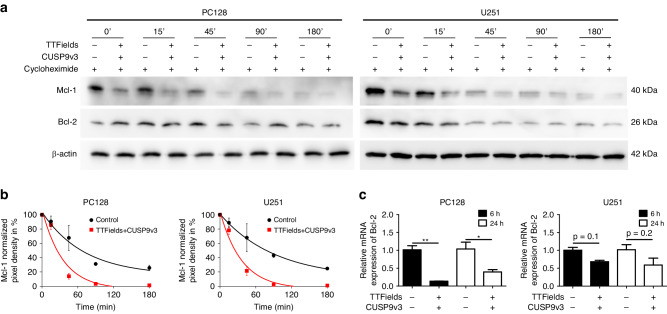
TTFields and CUSP9v3 combined lead to accelerated turnover of Mcl-1 protein and transcriptional downregulation of Bcl-2. **a** PC128 and U251 cells were subjected to treatment with solvent or TTFields/CUSP9v3 (PC128: 1/7.5 and U251: 1/2.5) for 24 h prior to exposing the cells to 10 µg/ml cycloheximide. At indicated time points, whole-cell extracts were collected. Western blot analysis was performed for the expression of Bcl-2 and Mcl-1. β-actin served as loading control. **b** PC128 and U251 cells were treated as described for (**a**). Densitometric analysis was performed using the Bio-1D software (Vilber Lourmat). Expression of Mcl-1 was normalized to β-actin and to the zero-time value of the respective treatment group in each independent experiment. Data are presented as mean and SD of three independent experiments. Mean data points were fitted to an exponential curve. **c** PC128 and U251 cells were treated for 6 h or 24 h as indicated. rt-PCR was performed for Bcl-2. Columns: mean of three technical replicates. Error bars: SD. *p < 0.05, **p < 0.01.

We next assessed the underlying mechanism of Mcl-1 and Bcl-2 downregulation by TTFields and CUSP9v3. In order to examine whether the protein stability of Mcl-1 or Bcl-2 is affected, glioblastoma cells were treated with cycloheximide in the presence or absence of the dual treatment. A comprehensive analysis was then conducted of the changes in Mcl-1 and Bcl-2 protein levels over time. Under the condition of blocked translation, simultaneous treatment with TTFields and CUSP9v3 accelerated the decay of Mcl-1 when compared to cells treated with solvents (Fig. 4a, b). Since the protein stability of Bcl-2 did not seem to be affected by the combination treatment, we next examined whether mRNA levels vary among cells treated with TTFields/CUSP9v3 and solvents. As shown in Fig. 4c, the dual treatment led to a marked decrease in *Bcl-2* mRNA expression as early as after 6 h of treatment and lasting for at least 24 h of treatment.

### Dual treatment with TTFields and CUSP9v3 suppresses oxidative phosphorylation

**Fig. 5:**
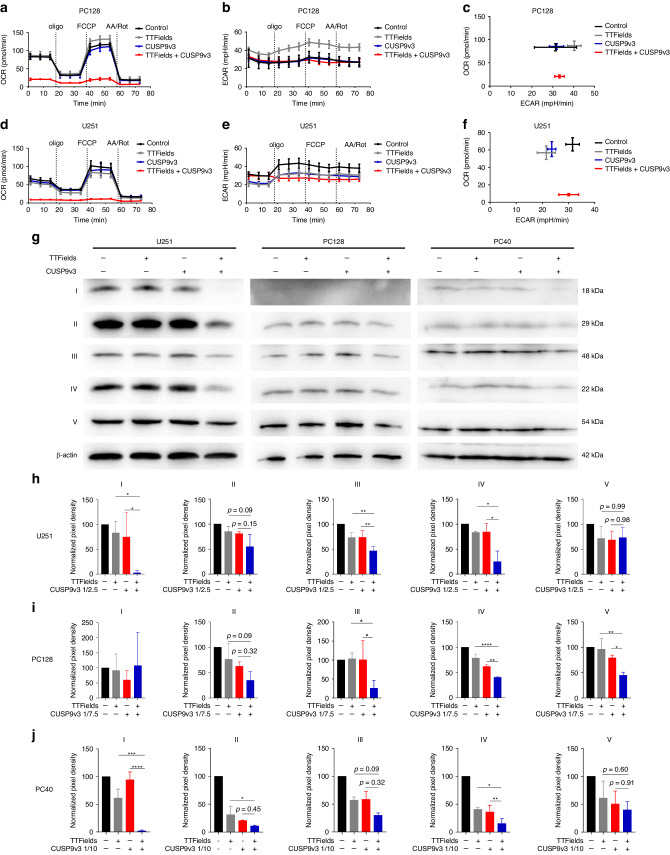
Combined treatment with TTFields and CUSP9v3 suppresses OXPHOS. PC128 (**a**) and U251 (**d**) glioblastoma cells were treated for 24 h as indicated. Mitochondrial stress tests were performed. Oxygen consumption rates (OCR) were continuously recorded while oligomycin (oligo), FCCP and antimycin A/rotenone (AA/Rot) were sequentially injected into the wells. Mean and SD of 4 technical replicates representative for 3 independent experiments. Extracellular acidification rates (ECAR) for PC128 (**b**) and U251 (**e**) cells treated as described for (**a**, **d**). Mean and SD of 4 technical replicates representative for 3 independent experiments. PC128 (**c**) and U251 (**f**) cells were treated for 24 h as indicated. Graphical representation of baseline OCR/ECAR-values. Mean and SD of 4 technical replicates representative for 3 independent experiments. **g** U251, PC128 and PC40 cells were subjected to treatment with solvent, TTFields, CUSP9v3 or the combination (U251: 1/2.5, PC128: 1/7.5 and PC40: 1/10) for 24 h. Whole-cell extracts were collected and Western blot analysis was performed for the expression of complex I–V of the respiratory chain. β-actin served as loading control. U251 (**h**), PC128 (**i**) and PC40 (**j**) cells were treated as described for (**g**). Densitometric analysis was performed using the Bio-1D software (Vilber Lourmat). Normalization to the respective β-actin signal and to control was performed. Data are presented as mean and SD of three independent experiments.

Since concomitant treatment with TTFields and CUSP9v3 significantly reduced the ATP content in glioblastoma spheroids (Fig. 1l–n), we next assessed whether this treatment also affects tumor cell metabolism. To this end, we performed extracellular flux analyses. In both, PC128 and U251 glioblastoma cells, oxidative consumption rates (OCR) were suppressed at baseline when compared to TTFields or CUSP9v3 alone (Fig. 5a, c, d, f). Moreover, during the course of mitochondrial stress tests OCRs remained at markedly reduced levels (Fig. 5a, d). In contrast, extracellular acidification rates were not altered in a consistent manner when compared to the single treatment modalities (Fig. 5b, c, e, f).

### Treatment with TTFields plus CUSP9v3 downregulates respiratory chain complexes I, III and IV

In order to further examine on a molecular level how the dual treatment inhibits the oxidative phosphorylation in glioblastoma cells, the expression of respiratory chain complexes was analyzed by Western blot (Fig. 5g–j). While the single therapeutic approaches led to some reduction of the expression of the proteins of the respiratory chain, the combination treatment induced significantly enhanced downregulation which was most consistently seen for complexes I, III and IV (Fig. 5g–j). For complex II, a similar trend was observed, however, statistical significance was not reached.

### The dual treatment inhibits the migration of glioblastoma cells

**Fig. 6:**
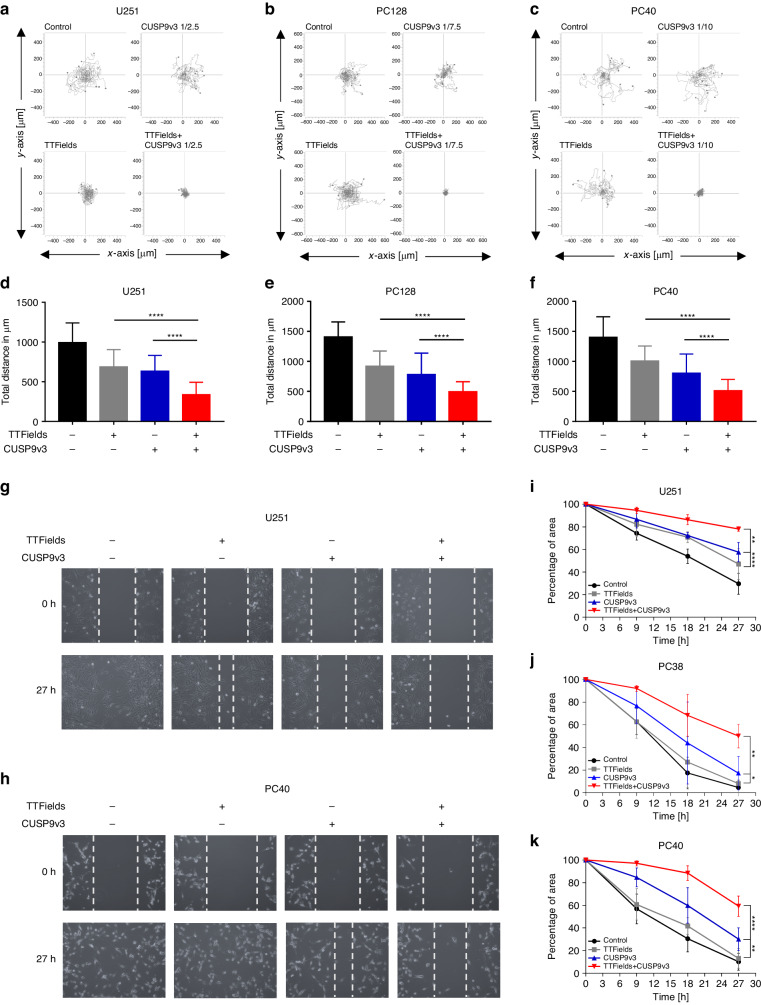
TTFields and CUSP9v3 combined have enhanced anti-migratory activity. U251 (**a**), PC128 (**b**) and PC40 (**c**) cells were treated for 24 h as indicated. Afterwards, the cells were enzymatically detached and seeded on 96-well plates followed by sequential microscopic imaging (magnification, × 10) over a total period of 24 h. Wind-rose plots displaying the paths of 15 single cells per treatment condition during the 24 h observation period. The tracks were aligned to start from the same initial position to facilitate comparison. **d**–**f** Total distance of 45 cells each tracked within 24 h per treatment condition. Columns, mean; bars, SD. Representative microphotographs of U251 (**g**) and PC40 (**h**) cells that were treated for 24 h as indicated followed by inflicting scratches across the subconfluent monolayers of these cells. Magnification, × 10. U251 (**i**), PC38 (**j**) and PC40 (**k**) cells treated as described for (**g**, **h**). Data are presented as mean and SD. *p < 0.05, **p < 0.01, ***p < 0.005.

Dysregulated migratory activity on one hand represents a typical feature of cancer cells and is highly energy-dependent on the other hand. We therefore tested whether the metabolic effects of the combination treatment are associated with reduced migration of glioblastoma cells. For this purpose, time lapse analyses were performed and wind rose plots were generated. As shown in Fig. 6a–c, the cellular tracks were less spread-out following treatment with the single therapeutic modalities while a strong restriction of the migratory paths was observed when the cells were treated with TTFields and CUSP9v3 together. Quantitative analysis showed a slight reduction of the total migratory distance following treatment with the single approaches which, however, was significantly more pronounced when the cells were subjected to the combination treatment (Fig. 6d–f). In order to examine whether this finding also holds true in other settings, scratch assays were performed (Fig. 6g–k). In line with the results of the time lapse analyses, treatment with TTFields or CUSP9v3 alone led to some inhibition of the closure of the scratch (Fig. 6g–k). However, when both treatment modalities were combined, the anti-migratory effects were significantly increased (Fig. 6g–k).

## Discussion

Drug repurposing has emerged as a promising translational approach to bypass the downsides of common drug development pipelines. Major advantages of repurposing older drugs are the lower costs of already existing drugs and a well-characterized safety profile [31]. Therefore, transition to clinical application is greatly facilitated. CUSP9v3 represents such an example which was conceptualized in 2013 [18], validated in the preclinical setting [21] and taken to application in patients, first in a compassionate use setting [21] and followed by a phase Ib/IIa clinical trial [22] in less than ten years and within a purely academic setting.

Expanding on one of the most important intrinsic principles of CUSP9v3, i.e., multitargeting, we have explored ways of enhancing the anti-glioblastoma activity of CUSP9v3 before. In a previous study, our data showed that CUSP9v3 combined with Bcl-2/Bcl-xL inhibition using Navitoclax (ABT-263) resulted in predominantly synergistic antineoplastic activity among different glioblastoma models [32]. In the present study, we observed that TTFields lower Bcl-2 and Mcl-1 levels, which may add to the biological effect when combined with CUSP9v3 similar to the mechanism seen when inhibiting Bcl-2/Bcl-xL by Navitoclax along with CUSP9v3 treatment. However, Bcl-xL levels were unaffected by TTFields or CUSP9v3 alone and by the combination which differs from the mechanism seen in the previous study. At this point, our data indicate that a reduction of Bcl-xL levels does not seem to be necessary to induce the biological effects of TTFields/CUSP9v3 in this setting. However, what we have not yet explored are the effects of the combination on the interaction with the natural pro-apoptotic counterplayers such as Bad, which may result in functional impairment of Bcl-xL. If Bcl-xL is not part of the mechanism, addition of a selective Bcl-xL inhibitor might further increase the efficacy of the TTFields/CUSP9v3 approach.

Notably, we observed that the antiglioblastoma activity following treatment with TTFields and CUSP9v3 was associated with suppression of oxidative phosphorylation. Alteration of the tumor cell metabolism as a prominent feature of cancer cells has first been described by Otto Warburg in the 1920s [33]. More recently, interest has grown in developing therapeutic strategies directly targeting tumor cell metabolism or approaches that take advantage of specific tumor cells’ metabolic vulnerabilities [34, 35]. Patel and colleagues recently reported that TTFields cause a shift away from the aberrant glycolytic metabolism in glioblastoma [36]. In that study, the authors used the radiotracer [^18^F]DASA-23 to measure pyruvate kinase M2 expression as a surrogate for the glycolytic activity in glioblastoma cells. In U87 cells, a 35% and in GBM39 cells, an 81% reduction in [^18^F]DASA-23 uptake was found following exposure to TTFields for 6 days. Our analyses also showed effects of TTFields alone or concomitant with CUSP9v3 on extracellular acidification rates as read-out for the glycolytic rate. However, we observed that these metabolic changes in response to TTFields were not consistent and varied among different cell models. The discrepancy between our work and the study of Patel et al. might in part be due to differences in timing. In this study, we examined earlier time points (after 24 h and 48 h of treatment), whereas Patel et al. examined the metabolic effects of TTFields after 72 h and 96 h. The varying results may also be attributed to differences in the surrogate markers, the different genetic background of the glioblastoma cells used and frequence specificity. In contrast, our analyses showed a very strong and consistent downregulation of OXPHOS when the cells were subjected to the combination treatment. To our knowledge, these are the first data showing suppression of OXPHOS following a treatment that includes TTFields. While we were not able to completely decipher the cause of this response, we noticed a significant decrease in the expression of respiratory chain complexes. Further studies are planned to gain more insight into the mechanism. Along this line, another tempting question is whether inhibition of additional metabolic pathways that are involved in the progression of glioblastoma, such as glycolysis, the pentose phosphate pathway or β-oxidation, might allow for further enhancement of TTFields/CUSPv3.

From a translational perspective, when applying drugs to target brain tumors, as in the CUSP9 approach, two important pharmacokinetic attributes need to be considered: First, the transfer of the drugs from the blood to the tumor and second, the distribution of the drugs within the tumor. Recently, Salvador et al. reported that TTFields may facilitate penetration across the blood-brain barrier [16]. In this study, the authors showed that application of TTFields at a frequency of 100 kHz for 72 h led to enhanced accumulation of Evans Blue and TRITC-dextran in rat brain tissue indicating increased permeability of the cerebral vasculature. Moreover, in an orthotopic rat glioblastoma model, treatment with paclitaxel, a drug that is known to poorly pass the blood-brain barrier, led to a significant reduction of tumor volume when combined with TTFields. Admittedly, other potential mechanisms besides a facilitated opening of the blood-brain barrier cannot be fully excluded. Along this line, another group observed that TTFields affect the structure of cancer cell membranes [15]. In this work, Chang et al. showed that exposure of U87-MG cells to TTFields at 200 kHz led to significantly enhanced cellular uptakes of ethidium D, 5-ALA and FITC-labeled dextran with sizes of 4 and 20 kDa. Moreover, electron microscopy showed that exposure to TTFields for 72 h led to a significantly increased number of holes in the membranes of U87MG cells. The results of these studies provide additional support of benefit from adding TTFields to treatment with CUSP9v3 in patients by enhancing exposure of glioblastoma cells to the CUSP9v3 drugs.

In conclusion, we provided proof of principle that a multimodal, multitargeting approach that includes TTFields and CUSP9v3 results in mutually enhanced activity against glioblastoma. Both treatment modalities on their own are mostly well-tolerated by patients and carry low risk for serious adverse events. These results encourage further studies and transition to the clinical setting.

### Supplementary information

Supplementary figure 1

## Data Availability

The data supporting the results reported in the article are deposited at the translational brain tumor research laboratory, Department of Neurological Surgery, Ulm University Medical Center, Albert-Einstein-Allee 23, D-89081 Ulm, Germany.
