# Supplementary material for: Tumor Treating Fields (TTFields) combined with the drug repurposing approach CUSP9v3 induce metabolic reprogramming and synergistic anti-glioblastoma activity in vitro
Source: Br J Cancer. 2024 Feb 23;130(8):1365–76. doi: 10.1038/s41416-024-02608-8 (PMC11015043; doi:10.1038/s41416-024-02608-8)
Supplement: Supplementary file 1 — Supplementary figure 1 [file 41416_2024_2608_MOESM1_ESM.pdf]

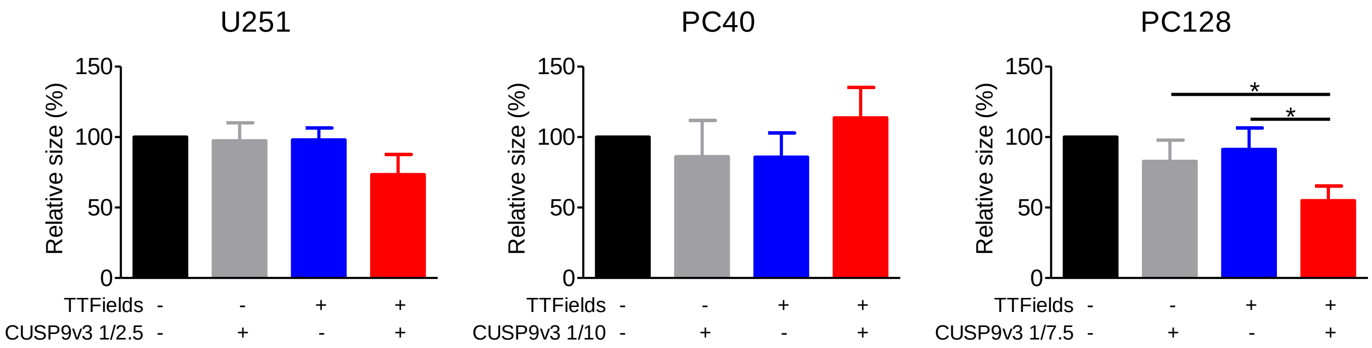

Supplementary figure 1:
Spheroids derived from U251, PC40 and PC128 cells were allowed to form for 5 d. Treatment as indicated was performed on d5, d7 and d9. On day 12, microphotographs were taken at 4 × magnification and maximal expansion of spheroids was determined. Data are presented as mean and SD of three independent experiments. *$p<0.05$.
